# Comparison of Two Doses of Ropivacaine Hydrochloride for Lumbosacral Epidural Anaesthesia in Goats Undergoing Laparoscopy Assisted Embryo Transfer

**DOI:** 10.1155/2014/937018

**Published:** 2014-09-25

**Authors:** Anubhav Khajuria, Mujeeb ur Rehman Fazili, Riaz Ahmad Shah, Firdous Ahmad Khan, Maajid Hassan Bhat, Syed Hilal Yaqoob, Niyaz Ahmad Naykoo, Nazir Ahmad Ganai

**Affiliations:** ^1^Division of Veterinary Surgery and Radiology, Faculty of Veterinary Science & AH, Shere-Kashmir University of Agricultural Sciences and Technology of Kashmir, Shuhama, Srinagar, Jammu and Kashmir 190006, India; ^2^Veterinary Clinical Service Complex, Faculty of Veterinary Science & AH, Shere-Kashmir University of Agricultural Sciences and Technology of Kashmir, Shuhama, Srinagar, Jammu and Kashmir 190006, India; ^3^Division of Animal Biotechnology, Faculty of Veterinary Science & AH, Shere-Kashmir University of Agricultural Sciences and Technology of Kashmir, Shuhama, Srinagar, Jammu and Kashmir 190006, India; ^4^Department of Animal Production, College of Food and Agricultural Sciences, King Saud University, Riyadh 11451, Saudi Arabia

## Abstract

Goats (*n* = 12) undergoing laparoscopy assisted embryo transfer were randomly allotted to two groups (I and II) and injected same volume of ropivacaine hydrochloride at 1.0 mg/kg and 0.5 mg/kg body weight, respectively, at the lumbosacral epidural space. The hind quarters of all the animals were lifted up for the first 3.0 minutes following injection. Immediately after induction the animals were restrained in dorsal recumbency in Trendelenburg position in a cradle. Laparoscopy was performed after achieving pneumoperitoneum using filtered room air. Regional analgesia and changes in physiological parameters were recorded. The mean induction time in animals of group I (*n* = 6) was 12.666 ± 1.994 minutes. In these animals the analgesia extended up to the umbilical region and lasted for 60 minutes. Only two animals in group II were satisfactorily induced in 11.333 ± 2.333 minutes. In animals of group I, the time taken for regaining the full motor power was significantly long (405 ± 46.314 min) when compared to group II goats (95 ± 9.219 min). From this study it was concluded that ropivacaine did not produce adequate analgesia in most of the goats at 0.5 mg/kg. When used at 1.0 mg/kg, it produced satisfactory regional analgesia lasting for one hour but the prolonged motor loss precludes its use. Additional studies using ropivacaine hydrochloride at doses in between the two extremes used here may be undertaken before recommending it for lumbosacral anaesthesia in goats undergoing laparoscopy.

## 1. Introduction

In ruminants, regurgitation and aspiration of ruminal contents or saliva into the lungs precludes the use of general anaesthesia [1, 2]. Epidural regional anaesthesia, being comparatively safe [3], is therefore the most frequently used technique before undertaking diagnostic, obstetrical, and surgical interventions particularly in sheep and goats [3, 4]. Easy availability at low price, lack of drug residues, excellent analgesia, and muscle relaxation are the additional advantages [2]. In small ruminants, laparoscopy has mainly been used in investigations related to the reproductive system. But most frequently these procedures have been performed after local infiltration of the portal sites along with sedation/tranquilization [5, 6]. Unlike local infiltration, epidural anaesthesia produces complete and homogenous analgesia of all the abdominal layers including highly painful peritoneum. The specific additional advantage during laparoscopy procedures would be the satisfactory muscle relaxation leading to increase in the intra-abdominal space and easier manipulations.

Ropivacaine hydrochloride, a long acting amide local anaesthetic agent [7], is well tolerated in human beings, reduces the potential for central nervous system and cardiac toxicities, and also lowers the propensity for motor blockade [8]. In a recent study, ropivacaine hydrochloride has been used at 0.6 mg/kg as lumbosacral epidural anaesthetic agent in goats, but these animals did not undergo any type of conventional or laparoscopic surgery after induction [9]. Perusal of the available literature shows that ropivacaine has till date not been used for inducing lumbosacral epidural regional anaesthesia in goats undergoing laparoscopy or laparoscopy assisted surgery. Keeping all the above-mentioned facts in mind, the study was planned with the objective of evaluating the effects of two doses of ropivacaine hydrochloride (keeping the volume constant) as lumbosacral epidural anaesthetic in goats undergoing laparoscopy assisted embryo transfer.

## 2. Material and Methods

The present study included twelve (*n* = 12) clinically healthy adult Pashmina and Bakarwal female goats (does) undergoing laparoscopy assisted embryo transfer. Standard management, feeding schedule, regularly deworming, and vaccination practices were followed in the farm to maintain all the animals. The study protocol was approved by the Institutional Animal Ethics Committee. Two days prior to the planned laparoscopy, the animals were thoroughly examined and their ventral abdomen clipped and shaved. They were fasted for 36–40 hours preoperatively. Drinking water was withheld for 18–20 hours only.

On the day of surgery, the does were assigned randomly to one of the two groups (I and II) and subjected to lumbosacral epidural anaesthesia. The animals were restrained in standing position against a concrete wall, while injecting epidural anaesthetic. A G-18 (3.85 cm length) disposable, sterile, fresh, hypodermic needle was used to inject the anaesthetic in every animal included in the study. The proper placement of the drug in the epidural space was ensured by adopting the “hanging drop” and “flow of the solution without resistance” methods. In goats of group I, ropivacaine hydrochloride (Ropin 0.5%, Neon Laboratories, India) was injected at 1.0 mg/kg body weight. Ropivacaine hydrochloride 0.5% was diluted with equal volume of normal saline (to keep the total volume constant) and injected at 0.5 mg/kg body weight in animals of group II. Immediately after epidural injection, the needle was withdrawn and the hind limbs of the animal lifted to 45° angle for the first three minutes. Subsequently the goats were allowed to stand on a level ground till induction.

After induction of regional anaesthesia, every goat was shifted to the operation theatre and restrained in dorsal recumbency in a cradle. The head region of the animal was lowered (Trendelenburg posture) making approximately 45° angle with the floor surface. Pneumoperitoneum using filtered air was created with the use of a Veress needle. Two ports (one at linea alba and the second 5.0–6.0 cm lateral to it) were created 3.0 to 3.5 cm cranial to the mammary glands, one for introduction of the laparoscope and the other for passing a grasping forceps. Detailed laparoscopic examination of the pelvic structures was conducted. One of the uterine horns was grasped; the midventral portal site enlarged longitudinally for evacuation of the abdominal air and to pull out the uterine horn for embryo transfer.

The onset, depth, and duration of analgesia and recovery were determined by noticing the sensory and motor responses in caudal body regions of the goats. Sensory responses of the area and/or animal to the pin pricks and or surgical interventions and manipulations were recorded in perineal, abdominal (5.0 cm cranial to mammary glands, over linea alba, and 5.0 cm lateral to it bilaterally), and umbilical (over linea alba and 5.0 cm lateral to it bilaterally) regions. Anal and hind limb pedal reflexes and tail analgesia were also evaluated. The reactions were graded on a 0 to 3 score scale where 0 represented no analgesia (strong reaction to pin pricks), 1 mild analgesia (weak response to pin pricks), 2 moderate analgesia (occasional response to pin pricks), and 3 strong/complete analgesia (no response to pin pricks) [10]. Motor deficit due to the lumbosacral regional anaesthesia was also graded on a 0 to 3 score scale—0: walking without staggering, 1: able to stand but walks with little ataxia/incoordination, 2: frequent swaying of the body but able to stand and walk with extreme incoordination, and 3: sternal recumbency/unable to stand [10]. The rectal temperature, heart and respiration rate, induction time, duration of surgery and pneumoperitoneum, and total duration of epidural anaesthesia were also recorded.

Clinical parameters and the analgesic scores were recorded immediately before giving lumbosacral epidural anaesthetic (*T*_0_) and immediately after satisfactory induction (*T*_1_), followed by every 15 minutes up to 1 hour and every 30 minutes up to 240 minutes and after every 60 minutes thereafter till complete recovery.

For the evaluation of analgesia score, Kruskal-Wallis test (nonparametric) was used for the comparison of (mean ± S.E) values. The data for clinical parameters were analyzed by ANOVA for repeated measures for comparison of (mean ± S.E) values between and within the groups using SPSS (20) version for Windows. Dunnett's *t*-test was used to determine time at which treatment response differed from baseline. The values of the clinical parameters were considered significant at *P* < 0.05.

## 3. Results

The weight and age of the animals included in this study have been presented in Table 1. The age and weight (mean ± SE) of the goats belonging to group I were 21.000 ± 1.341 months and 30.500 ± 1.565 kg and those of group II were 22.00 ± 1.264 months and 33.000 ± 1.095 kg, respectively. There was no significant difference between the values of age and body weight of the goats belonging to the two groups.

Location of the lumbosacral space in standing animals was easy as the landmarks for its identification were specifically followed. Restraining the animal adjacent to a concrete wall prevented sideway movement of the animal during the placement of the needle into the epidural space and deposition of the local anaesthetic. Use of G-18 hypodermic needle and following the hanging drop and no resistance techniques for accurate deposition of the anaesthetic were successful in all of the animals.

The analgesic scores of the various caudal body regions obtained in animals of both the groups at various time intervals have been presented in Figures 1, 2, 3, 4, 5, 6, 7, 8, 9, and 10.

In animals of group I, the analgesia of all the areas from tail to the umbilical region was complete (score 3) from *T*-1 to *T*-60 interval. It started declining from T-90 onwards. Moderate analgesia (score 2) was present in these goats up to T-180. The analgesia vanished completely (score 0) at *T*-540.

In most of group II animals, only mild analgesia (score 1) developed in anal, tail, perineal, and right umbilical areas up to time interval *T*-30. Although left paramedian abdominal area showed satisfactory analgesia (score 3) up to *T*-15; but umbilicus, left side of the umbilicus, and rest of the abdominal wall showed moderate (score 2) analgesia up to *T*-30. In this group sensory analgesia vanished completely at *T*-90. In four of the six animals of this group, the analgesia of the portal sites had to be supplemented by the use of 1% lignocaine hydrochloride (1.0 mL/site) by local infiltration.

The bilateral hind limb motor power returned to normal level in 405 ± 46.314 minutes and 95 ± 9.219 minutes in animals of groups I and II, respectively. The difference between the groups was statistically significant (*P* < 0.05).

The mean (±S.E) induction time in animals of group I was slightly and insignificantly longer (12.666 ± 1.994 minutes) than those belonging to group II (11.333 ± 2.333 minutes). The duration (mean ± S.E) of pneumoperitoneum in group I and II goats was 13.166 ± 2.535 minutes and 14.833 ± 2.072 minutes, respectively. The surgical time (mean ± S.E) was 42.500 ± 7.539 and 38.333 ± 7.264 minutes, respectively. All of the values pertaining to the above-mentioned parameters did not differ significantly (*P* > 0.05) between the groups.

The values (mean ± S.E) of various physiological parameters obtained in animals of both the groups up to *T*-150 have been presented in Table 1. The respiration rate did not show any significant (*P* > 0.05) difference within or between the groups. Animals of group II showed significantly (*P* < 0.05) higher rectal temperature values at *T*-30, *T*-45, and *T*-60 but significantly (*P* < 0.05) lower heart rate value at *T*-45 than the corresponding values in animals of group I. The heart rate was significantly higher up to *T*-150 in goats of group I when compared to their base value but showed no significant difference with the corresponding values in goats of group II.

## 4. Discussion

Ropivacaine has been tried in this study for the first time in normal goats undergoing laparoscopy assisted embryo transfer. The animals of the two breeds were randomly and equally distributed and their age and body weights were almost similar.

The restraint of the animal against a wall in standing position resulted in easy deposition of the local anaesthetic in the lumbosacral epidural space. The procedure was accomplished with minimal man power requirement and without distress to the animal. Lateral decubitus with the lumbosacral spine in full flexion has been used as an alternative way of restraint for effective deposition of lumbosacral anaesthetic agents in small ruminants [11–13].

The standard dose of lignocaine hydrochloride, the most frequently used epidural anaesthetic in small ruminants, is 4.0 mg/kg [13]. Ropivacaine hydrochloride is four times more potent than lignocaine [13]. A dose of 1.0 mg/kg ropivacaine was therefore considered to be equipotent to 4.0 mg/kg lignocaine hydrochloride. In animals of group I, the dose rate was decided accordingly. The dose of ropivacaine hydrochloride used in goats of group II of our study was 0.5 mg/kg. Ropivacaine has also been used for perioperative epidural analgesia at 1.0 mg/kg and 0.5 mg/kg in dogs undergoing tibial plateau levelling osteotomy [14]. In goats, it has been used as 0.6 mg/kg, but these animals were not subjected to any type of surgery [9, 15, 16]. Lower dose (0.1 mg/kg) has been used for caudal epidural block in cattle [17].

Before infusing the anaesthetic agent in group II animals, normal saline was added to the calculated dose of anaesthetic preparation (50 : 50) for keeping the volume of the anaesthetic preparation constant with that of group I goats. Physiological normal saline has been used to adjust the volume of the spinal anaesthetic in goat [12]. Many factors have been shown to affect the cranial spread of anaesthetic within the epidural space. The variables that can be controlled by the anaesthetist are, namely, positioning the patient, choosing the site of epidural puncture, orientation of the needle bevel, determining the volume and concentration of the anaesthetic solution, and speed of injection [18].

The actual surgical/laparoscopic intervention used in this study is the ideal procedure to evaluate quality of analgesia. In all the earlier studies wherein ropivacaine was used for epidural anaesthesia in goats, the sensory analgesia was evaluated on the basis of the response of the animal to percutaneous pin prick stimulation [9, 15, 16].

Sterile, fresh, and hypodermic needles used in the goats of this trail to inject anaesthetic preparation are not only cheap but also easily available in sterile packing. The diameter and the length of the needles (G18, 3.85 cm) were sufficient to deposit the local anaesthetic in the epidural space in all the goats. Shorter (3.25 cm or 3.50 cm) hypodermic needles (G18 or G20) have also been used for epidural [12, 19] and subarachnoid [20] analgesia in small ruminants. In fat-tailed sheep with mean (±S.E) weight 24.6 ± 2.5 kg (range 21 kg–27 kg), the mean distance from the skin to the epidural space has been found 3.4 ± 0.3 cm (range 3.0 cm to 3.9 cm) by earlier workers [21]. However, the spinal needles having a blunt tip and a stylet within its lumen are used in most of the species conventionally [11].

It has been propounded that when the animal is allowed to stand on a level surface, analgesia extends only up to a fourth of the distance from the pubis to the umbilicus [21]. According to the earlier reports, ropivacaine produced analgesia of tail, perineum, inguinal, and thigh regions only in goats induced lumbosacral epidural anaesthesia [9, 14, 15]. More cranial extension of the analgesia simultaneously from tail to the umbilical area recognized in animals of this study might have resulted from gravity during induction and due to dorsal recumbency and Trendelenburg position during laparoscopy. It has been recognized that keeping the patient in a head-up or head-down position may affect the spread of local anaesthetic solution within the epidural space [22].

The time to onset of analgesia of group I animals of this study was longer than the value reported in goats [9] but similar to cattle subjected to caudal epidural anaesthesia [17]. The cranial spread of the anaesthetic agent within spinal canal due to lifting of the hindquarters of the goats followed by Trendelenburg posture might be responsible for this delay.

The animals of the two groups were subjected to a short period (range: 10–17 minutes) of pneumoperitoneum. They were kept in Trendelenburg position during this period. The position of Trendelenburg is convenient for cases in which it is desirable to allow the abdominal viscera to gravitate towards the diaphragm and thus facilitate visualization and manipulations in the pelvic region [22].

The anal and hind limb pedal reflex were absent and the tail and perineal analgesia profound during initial 60 minutes after induction in animals of group I. Similar duration of analgesia in the these areas was noticed in healthy goats earlier [15]. The reflexes and responses to sensory stimulation returned to normal level after nine hours in animals of group I but only after one and a half hours in group II goats. Full perineal sensation returned after six hours following caudal epidural block with ropivacaine (0.1 mg/kg) in cattle [17]. In animals of group II, satisfactory analgesia did not develop any of the above-mentioned areas. Inadequate analgesia was also noticed in dogs when ropivacaine was used at 0.5 mg/kg [14].

The analgesia in the umbilical and abdominal areas was satisfactory for one hour in those injected 1 mg/kg ropivacaine hydrochloride (group I). The mild analgesic effects also persisted for a long period (seven hours at umbilicus and eight hours in the abdominal region). In animal's injected lower dose of ropivacaine hydrochloride (group II), only mild analgesia developed in the umbilical and midventral and right paramedian abdominal areas. However, satisfactory analgesia for a brief period (15 minutes) was detected in the midventral to left paramedian abdominal area only. In these animals the local infiltration of low dose of lignocaine after this period extended analgesia of the ports for the required period. Analgesia beyond flank region did not develop in goats following lumbosacral epidural anaesthesia using ropivacaine when the animals were not maintained in head-down posture after injection of the anaesthetic [9, 15].

The bilateral anaesthesia is possible if the animal is positioned prone or supine so that the vertebral canal is horizontal [22]. In animals of group II, surprisingly the analgesia developed in the abdominal and umbilical areas was better than the caudal body areas for a brief period. The variation in the magnitude of analgesia between caudal and cranial areas and the two sides of the abdomen noticed in animals of this group might have resulted from the occasional voluntary movement and change in the posture of two animals before infiltration of the portal sites with local anaesthetic.

The rectal temperature and respiration rate values in both the groups were similar before injecting anaesthetic. These values also matched well with the values reported by several workers in normal goats [3, 12, 23]. However, higher values were reported in another study [19]. Subsequently there was a significant increase in the mean rectal temperature values in animals of group II from 15 minutes to 60 minutes after induction when compared to the corresponding values of the animals belonging to group I. Higher ambient temperature or distress signs revealed by two goats of this group might be responsible for this increase in temperature [24]. Increase in the rectal temperature attributed to the higher ambient temperature following epidural ropivacaine has also been noticed in cattle [17]. But nonsignificant decrease in the rectal temperature has been noticed in goats [9, 15]. Higher muscle activity and metabolism of individual goats may also lead to increase in the body temperature [25]. The animals of group I showed significant increase in the heart rate values from induction to 150 minutes than their base value. However, these values were still within normal range. Increase in heart rate may occur due to stress [26] induced by pneumoperitoneum and Trendelenburg posture in the animals of our study as tachycardia has not been reported after epidural anaesthesia with ropivacaine in goats not subjected to any type of surgery [9, 15]. However, supplementation of a tranquilizer/sedative along with epidural anaesthesia in goats undergoing laparoscopy needs to be evaluated in future. In group II animals, supplementation of the anaesthesia by local infiltration in four of the six goats may be responsible for keeping the heart rate persistently closer to the base value.

## 5. Conclusions

From this study, it was concluded that ropivacaine at 0.5 mg/kg as a lumbosacral epidural anaesthetic agent in goats undergoing laparoscopy did not produce satisfactory regional anaesthesia. When used at 1.0 mg/kg, satisfactory regional anaesthesia developed at least for one hour. However, its use at this dose may not be recommended due to the undesirable prolonged delay in regaining hind limb motor activity.

## Figures and Tables

**Figure 1:**
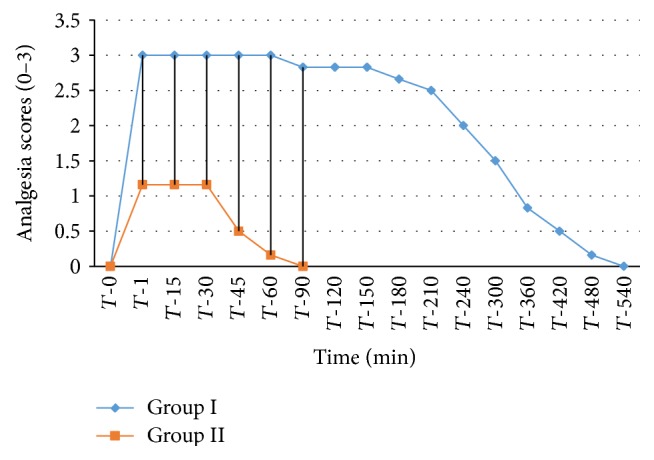
Loss and return of anal reflex in goats subjected to lumbosacral anaesthesia using two doses of ropivacaine.

**Figure 2:**
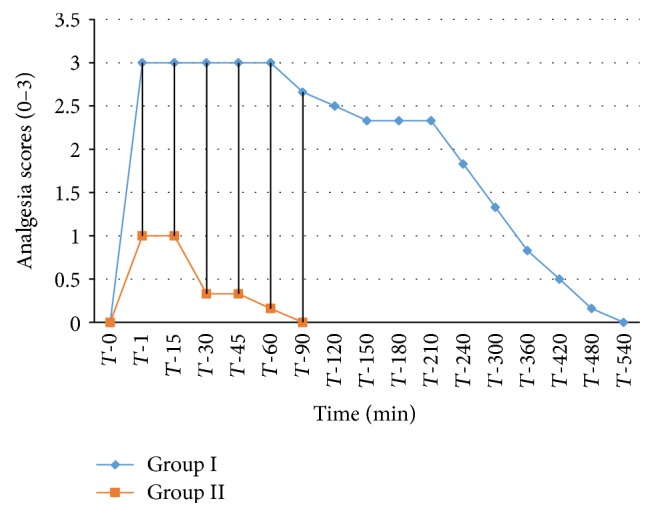
Tail analgesia in goats subjected to lumbosacral anaesthesia using two doses of ropivacaine.

**Figure 3:**
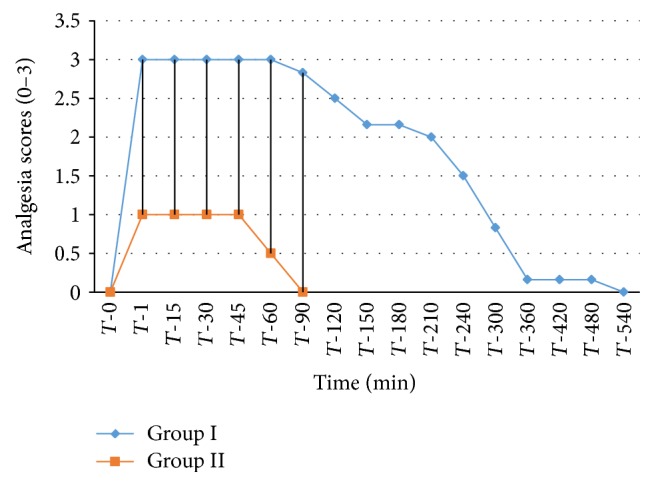
Analgesia in perineal region in goats subjected to lumbosacral anaesthesia using two doses of ropivacaine.

**Figure 4:**
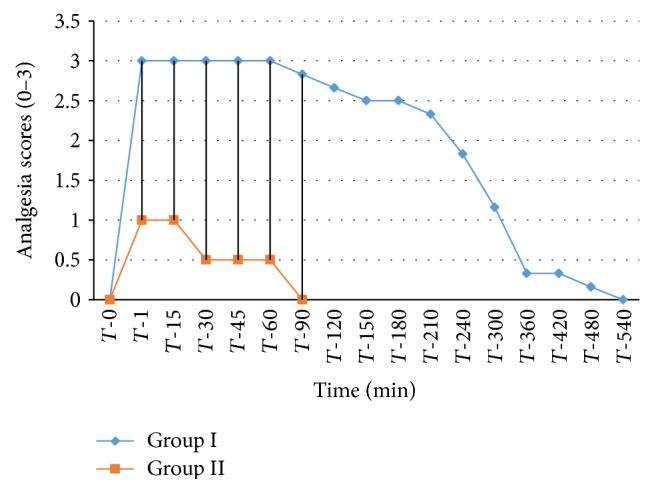
Loss and return of pedal reflex in goats subjected to lumbosacral anaesthesia using two doses of ropivacaine.

**Figure 5:**
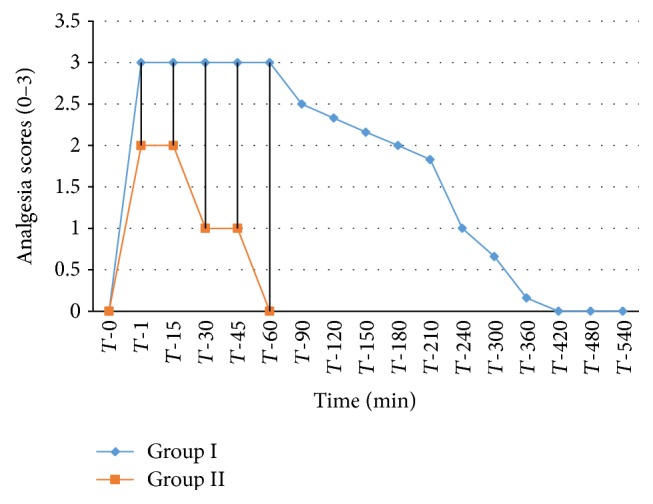
Analgesia of the umbilicus in goats subjected to lumbosacral anaesthesia using two doses of ropivacaine.

**Figure 6:**
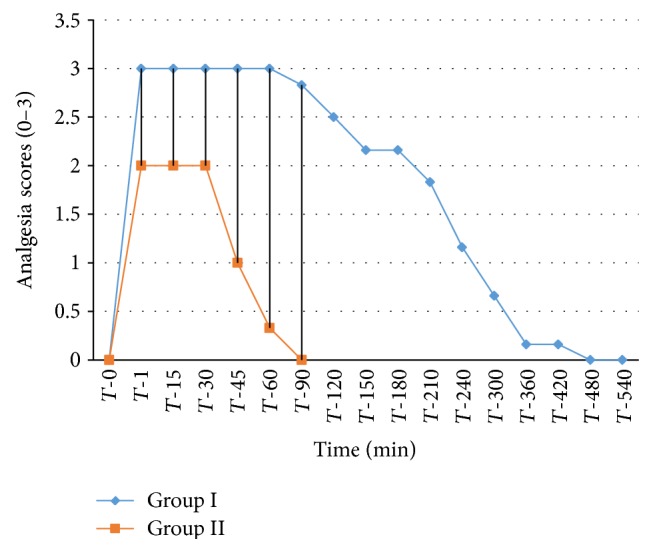
Analgesia in left paraumbilical area in goats subjected to lumbosacral anaesthesia using two doses of ropivacaine.

**Figure 7:**
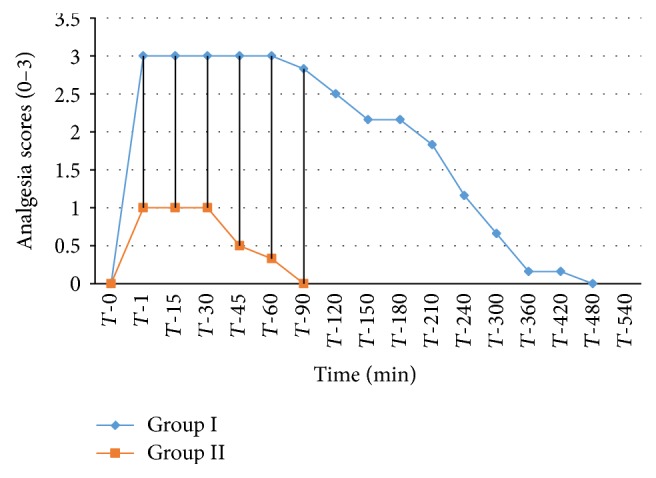
Analgesia in right paraumbilical area in goats subjected to lumbosacral anaesthesia using two doses of ropivacaine.

**Figure 8:**
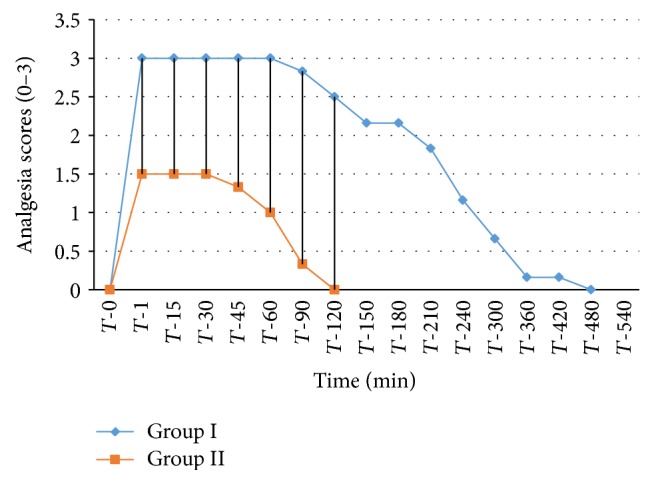
Analgesia in midventral abdominal wall in goats subjected to lumbosacral anaesthesia using two doses of ropivacaine.

**Figure 9:**
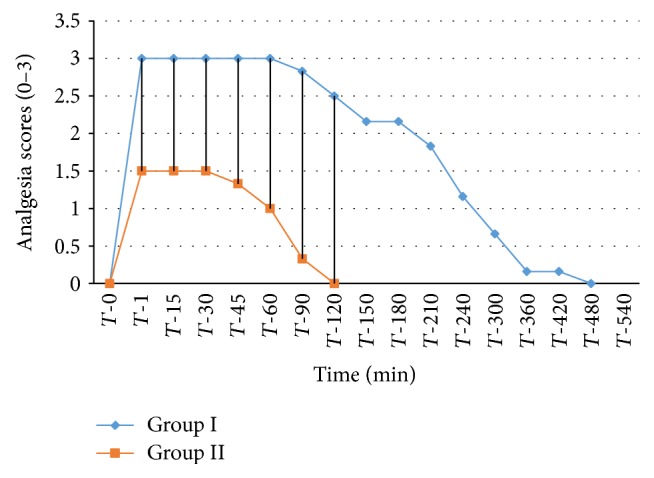
Analgesia of right abdominal wall in goats subjected to lumbosacral anaesthesia using two doses of ropivacaine.

**Figure 10:**
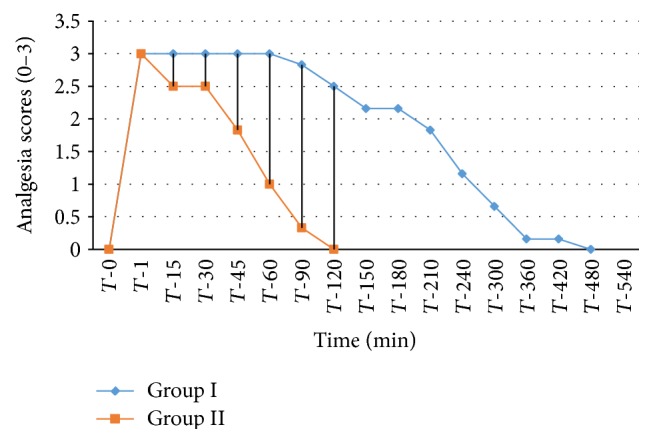
Analgesia of left abdominal wall in goats subjected to lumbosacral anaesthesia using two doses of ropivacaine.

**Table 1:** Comparative physiological values (mean ± S.E) up to *T*-150 in goats injected two doses of ropivacaine HCl as lumbosacral epidural anaesthetic.

	Group	Time								
		*T*-0	*T*-1	*T*-15	*T*-30	*T*-45	*T*-60	*T*-90	*T*-120	*T*-150
Rectal temperature (°F)	I	101.66 ± 0.16	102.33 ± 0.37^a^	102.11 ± 0.33^a^	102.11 ± 0.33^a^	101.95 ± 0.30^a^	101.93 ± 0.30^a^	101.46 ± 0.30	101.53 ± 0.31	101.81 ± 0.22
	II	101.91 ± 0.17	103.30 ± 0.24^b^	103.20 ± 0.28^b^	103.20 ± 0.22^b^	103.18 ± 0.20^b^	103.04 ± 0.24^b^	102.65 ± 0.45	102.20 ± 0.24	N.D

Respiration rate (breaths/min)	I	23.88 ± 1.97	36.66 ± 12.75	38.33 ± 12.48	40.83 ± 11.71	36.66 ± 10.10	39.66 ± 11.10	31.33 ± 10.55	31.66 ± 8.92	33.00 ± 9.51
	II	24.50 ± 2.75	43.33 ± 8.58	47.00 ± 8.35	44.33 ± 4.20	42.66 ± 3.78	33.20 ± 2.15	28.00 ± 0.00	26.00 ± 0.00	N.D

Heart rate (beats/min)	I	68.00 ± 6.92^A^	78.16 ± 5.96^BC^	82.33 ± 7.27^BC^	88.66 ± 4.75^BC^	86.66 ± 4.21^aBC^	95.33 ± 11.56^C^	80.66 ± 2.17^BC^	77.00 ± 2.90^BC^	76.00 ± 4.13^BC^
	II	68.33 ± 5.90^A^	68.00 ± 8.26^A^	77.00 ± 7.99^A^	71.00 ± 9.48^A^	69.00 ± 7.81^bA^	72.20 ± 11.56^A^	75.00 ± 14.00^A^	76.00 ± 8.40^A^	N.D

Figures with different superscript (small letters) differ significantly between the groups.

Figures with different superscript (capital letters) differ significantly between time intervals within the groups.

N.D: not determined.
